# A Comparison of Pharyngeal Swabs and Tracheal Secretions for the Diagnosing of COVID-19

**DOI:** 10.3390/biomedicines10020488

**Published:** 2022-02-18

**Authors:** Maibritt Meldgaard Arildsen, Sif Bay Glenting, Anette Marianne Fedder, Bettina Jørgensen, Svend Ellermann-Eriksen, Marianne Kragh Thomsen, Christina Catherine Dahm, Michael Pedersen

**Affiliations:** 1 Comparative Medicine Lab, Department of Clinical Medicine, Aarhus University, 8200 Aarhus, Denmark; 201709669@post.au.dk (M.M.A.); 201307348@post.au.dk (S.B.G.); 2 Emergency Department, Aarhus University Hospital, 8200 Aarhus, Denmark; anetfedd@rm.dk; 3 Department of Renal Medicine, Aarhus University Hospital, 8200 Aarhus, Denmark; bettina.stolle@auh.rm.dk; 4 Department of Clinical Microbiology, Aarhus University Hospital, 8200 Aarhus, Denmark; svenelle@rm.dk (S.E.-E.); marthoms@rm.dk (M.K.T.); 5 Research Unit for Epidemiology, Department of Public Health, Aarhus University, 8000 Aarhus, Denmark; ccd@ph.au.dk

**Keywords:** COVID-19, SARS-CoV-2, pharyngeal swab, tracheal suction, test methods, comparison

## Abstract

The aim of this study was to compare the test results from patients who, within a short timescale, have been tested for COVID-19 using both a pharyngeal swab and tracheal secretion. Data were collected from the database of AUH, from patients hospitalized between 1 March 2020 and 1 March 2021 who, due to symptoms of COVID-19, were tested by a pharyngeal swab and by tracheal secretion. We found great agreement between oropharyngeal swab and tracheal secretion RT-PCR testing for the diagnosis of COVID-19, with 98.5% of double tests being concordant and only 1.5% being discordant. This finding may advocate a single-test strategy being either an oropharyngeal swab RT-PCR testing or tracheal secretion, although this study revealed 15.9% false negative oropharyngeal swabs.

## 1. Introduction

In February 2020, the first Danish patient with COVID-19 was diagnosed, and due to the extensive transmission of the virus SARS-Cov-2, Denmark was within a few weeks widely affected by the COVID-19 pandemic [1,2]. Consequently, testing for SARS-CoV-2 by PCR was promptly established by the Danish Health Authority, because rapid and reliable testing is essential in controlling the COVID-19 pandemic. 

Several test methods for the detection of SARS-CoV-2 are used worldwide, however nucleic acid-based tests are considered the gold standard methods [3]. Additionally, different types of clinical specimens are usable for the detection of SARS-CoV-2 [4]. Therefore, when recommending a test method for the diagnosis of COVID-19, both the analysis procedure and specimen collection should be considered.

**Figure 1:**
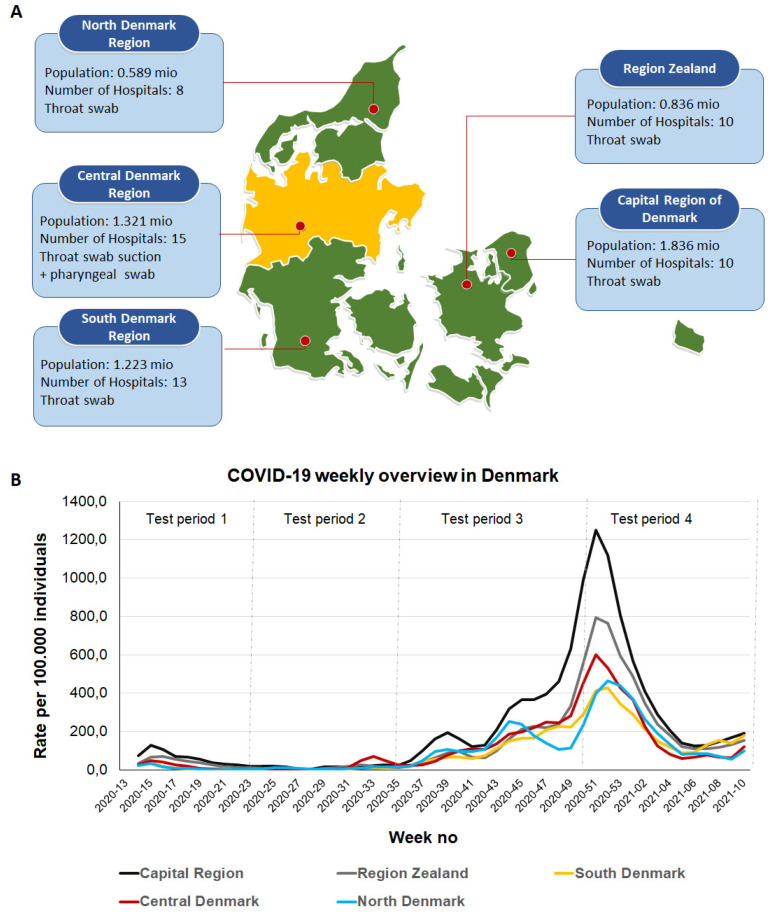
(**A**) Danish regional guidelines for the COVID-19 test-based diagnostics of hospitalized symptomatic patients. (**B**) COVID-19 weekly incidence. Adapted from https://www.ecdc.europa.eu (accessed on 27 December 2021); European Centre for Disease Prevention and Control (ECDC), 2022.

Pharyngeal swab RT-PCR testing from the upper respiratory tract or tracheal suction RT-PCR testing from the lower respiratory tract is often used as the primary diagnostic method, because they yield early results with moderate sensitivity (57.9%) and excellent specificity (94.6%) [5,6,7]. The testing strategy for COVID-19 often takes each country’s or region’s specific context into account. In Denmark, the Public Healthcare System is largely administrated by 5 regions, each governed by regional councils. Notably, a single region (Central Denmark Region) has, since March 2020, issued instructions about COVID-19 tests, including parallel oropharyngeal swab and tracheal suction in each patient presenting symptoms of COVID-19; as opposed to the other 4 Danish regions that have issued tests based on one of these methods only (Figure 1A) [8].

The implementation of parallel testing raised discussions of both healthcare resources and economical questions. Our aim was to compare pharyngeal swab and tracheal suction for the clinical diagnosis of COVID-19 in consecutive patients admitted to a single-site hospital with suggestive symptoms, and to determine the degree of heterogeneity among subgroups.

## 2. Materials and Methods

This retrospective study included patients hospitalized at Aarhus University Hospital (Central Denmark Region, Aarhus, Denmark) between 1 March 2020 and 1 March 2021 who presented symptoms of COVID-19. Patients were eligible for inclusion if they had reportable SARS-CoV-2 RT-PCR results from material obtained by both a pharyngeal swab and tracheal secretion. The two tests should be undertaken within a time difference of less than 12 h. Patients were excluded if they presented test results that were either inconclusive, unsuccessful or inhibited, or if aged < 18 years. 

We identified 4204 patients from registry search who met the inclusion criteria, providing a total of 4313 double tests. From these data, we excluded 909 double tests because specimens collected by the pharyngeal swabs and tracheal suctions were mixed during laboratory analyses without separate analyses. As result, a study population of 3315 patients, from whom 3404 double tests were included in this study. 

The following data were collected for each patient: personal ID, patient ID, age, gender, the unit of hospitalization, diagnosis ID, date of hospitalization, date of discharge, type of swab, date and time of the swab, type of tracheal secretion (tracheal secretion, secretion from the airways, aspirate/suction, or expectorate), date and time of the tracheal secretion, and tests results of both tests (based on RT-PCR analyses).

The dataset is presented in table format and statistical analyses were performed with χ^2^ tests.

## 3. Results

Demographics are presented in Table 1.

By comparing the distribution of concordant and discordant test results for genders, a χ^2^ value at 2.74 was found. The corresponding *p*-value was 0.098, suggesting no statistical differences between gender and the distribution of concordant and discordant tests results. Further, we found no correlation between the age of patients and the probability of receiving a discordant result. The distribution of results of the double tests was grouped in 4 periods, and interestingly, most discordant test results were collected during the last period. In total, 76.0% of the discordant test results were collected during the 4th period. To examine whether the distribution of test results was independent of the period for collection, we found a statistical χ^2^ value of 149.2 and corresponding *p* < 0.001, suggesting a positive correlation between the period of double testing and the results of the double tests.

The test results of the pharyngeal swabs and the tracheal secretions are compared in Table 2, demonstrating that 98.5% of the double tests showed congruent test results and 1.5% of the double tests showed discordant results.

The tentative clinical diagnoses of those registered with discordant test results are described in Table 3, illustrating that these patients mostly were registered with tentative diagnoses related to COVID-19.

## 4. Discussion and Conclusions

We found great agreement between the test results of oropharyngeal swabs and tracheal secretions collected from patients with symptoms of COVID-19, with only 1.5% of the double tests being discordant. We found no associations between the patients with discordant test results and their gender or age. Surprisingly, we found that most of those with discordant test results (70%) were hospitalized within the 4th period of the study (2 December 2020–1 March 2021). During the first period of the study (1 March 2020–1 June 2020) the daily incidence of COVID-19 in the Danish population was 30–390 positive cases per day, whereas the daily incidence in the 4th period of the study was 390–4500 positive cases per day. Fig 1B shows graphically the weekly incidence during the 4 study periods for all 5 Danish regions, demonstrating a significant increase in the incidence ratio per 100,000 individuals in test periods 3 and 4 compared to test periods 1 and 2. This difference in incidence ratios might contribute to the explanation of our finding with most discordant test results in the 4th period [2]. However, the proportion of discordant double tests registered in the 4th period (76%) is percentage-wise larger than the proportion of double positive test results in the period (51.2%), which may indicate that the increasing incidence of COVID-19 in Denmark during the 4th period cannot explain this finding alone. Additionally, it should be considered that mutations of the SARS-CoV-2 virus are developing during the pandemic, thus affecting the sensitivity and specificity of the RT-PCR analysis over time [9,10,11,12,13]. In Denmark, the original SARS-CoV-2 virus strain was dominating during most of our study period (1st March 2020–1st March, 2021), however the alpha-subtype of the virus strain was registered in November 2020, and it became the dominating SARS-CoV-2 strain in March 2021 [14]. Furthermore, single mutations of the virus strain have been registered continuously during the pandemic [14]. The increase of discordant test results in the 4th period of the study coincides with the progression of the alpha-subtype in Denmark, and should therefore be considered as a possible explanation of our finding most discordant test results in the 4th period of the study.

The found agreements between the test results of RT-PCR testing of pharyngeal swabs and tracheal secretions largely supports previous findings. Nazerian et al. documented that laryngotracheal aspiration RT-PCR testing in addition to nasopharyngeal RT-PCR testing reduced the false negative rate compared to nasopharyngeal RT-PCR testing as the only test modality [15]. However, this reduction required 10 aspirations to detect 1 false negative nasopharyngeal swab. This finding is in line with our results, demonstrating 15.9% false negative oropharyngeal swabs. Furthermore, Ünsaler et al. documented a great agreement between nasopharyngeal swab and nasopharyngeal aspiration RT-PCR testing for the detection of COVID-19 [16].

The found results may raise concerns about the parallel use of pharyngeal swab and tracheal suction as part of institutional/regional/governmental guidelines, including test procedures that are expensive, time-consuming, and uncomfortable for the patient. The purpose of double testing of these patients is to immediately analyze secretion from both the upper and lower respiratory tract when a patient is suspected with COVID-19 [8]. A recent study demonstrated that the test results of COVID-19 positive patients depend on the extent of symptoms (mild or severe) and the area of the respiratory tract where test material is collected (upper or lower) [17]. This finding emphasizes the importance of collecting material from the area of the respiratory tract that is infected. Additionally, different correlations between the SARS-CoV-2 viral load over the course of infection and the area of specimen collection (upper or lower respiratory tract) have been found [18]. However, our results showed great agreement between the results of the double tests from the upper and lower respiratory tract, with only 1.5% of the double tests being discordant. The explanation of this finding could be that 1.5% of the patients presented with different viral loads in the upper and lower respiratory tract, but other potential explanations could be different sensitivities of the two tests, or that both tests have the same sensitivity and miss true cases at random.

The RT-PCR cycle threshold (Ct) values are demonstrated to be inversely proportional to the viral load of SARS-Cov-2, and furthermore associated with the transmissibility of the virus [12,19]. When performed by the Danish Test system, the Ct cut-off value of the RT-PCR analysis is defined as 38 cycles [20]. Patients with low viral loads might present with Ct-values around the cut-off value, which could also precipitate the occurrence of some discordant tests. 

When evaluating the test procedures with clinical staff performing pharyngeal swabs and tracheal suctions, it becomes clear that it is clinically difficult to reach an appropriate depth of the respiratory tract, and thus, it should be questioned if materials that are registered as tracheal secretion more correctly represent secretion from the lower pharynx. With that perspective, it should be considered if the explanation of the agreement of our results is that both procedures reach secretion from closely related areas of the respiratory tract. Results reported by Ünsaler et. al. emphasized this clinical suspicion, demonstrating a great agreement in RT-PCR test results acquired from nasopharyngeal aspiration and nasopharyngeal swab collected simultaneously from hospitalized patients suspected with COVID-19 [16]. Another factor that contributes to a false-negative test result is the time point where the test is collected during the course of infection [21,22], because patients with suspected COVID-19 are likely in a later disease stage than patients without symptoms, and the abundance of discordant findings shown in Table 3 are related to patients registered with a COVID-19 diagnosis code.

The aim of this project was to compare pharyngeal swab and tracheal suction for the clinical diagnosis of COVID-19 in consecutive patients admitted to a single-site hospital with suggestive symptoms, and to determine whether there was heterogeneity across subgroups. We found great agreement between the two test methods, with only 1.5% of the double tests being discordant. We found no correlation between patients with discordant test results and their gender and age, but we found that most patients with discordant test results were hospitalized during the 4th period of our study, and that these patients mostly were registered with tentative diagnoses associated to COVID-19. The found differences over the study periods is likely explained by an increased incidence of COVID-19 in Denmark during the 4th period simultaneously with the progression of the alpha-subtype of the SARS-CoV-2 strain in Denmark. In conclusion, we found great agreement between oropharyngeal swab and tracheal secretion RT-PCR testing for the diagnosis of COVID-19, with 98.5% of double tests being concordant and only 1.5% being discordant. This finding may advocate a single-test strategy being either an oropharyngeal swab RT-PCR testing or tracheal secretion, although this study revealed 15.9% false negative oropharyngeal swabs.

## Figures and Tables

**Table 1:** Patient characteristics (gender, age) grouped in test results (positive/negative COVID result, pharyngeal swab/tracheal suction).

Pharyngeal Swab		Positive	Negative	Positive	Negative
Tracheal Secretion		Positive	Negative	Positive	Negative
		*n*: Number of patients (number of tests)			
		%: Percent of patients (percent of tests)			
**Males**		80 (98)	1677 (1705)	9 (9)	11 (12)
		4.5 (5.4)	94.4 (93.5)	0.5 (0.5)	0.6 (0.7)
	**Age (years)**				
	<30	3 (3)	97 (99)	0 (0)	1 (1)
	30–40	3 (4)	99 (101)	0 (0)	1 (1)
	41–50	12 (12)	116 (118)	0 (0)	2 (2)
	51–60	15 (25)	203 (208)	1 (1)	0 (0)
	61–70	24 (27)	334 (344)	5 (5)	1 (1)
	71–80	14 (18)	472 (479)	2 (2)	4 (5)
	81–90	8 (8)	286 (286)	1 (1)	2 (2)
	91–100	1 (1)	69 (69)	0 (0)	0 (0)
	>100	0 (0)	1 (1)	0 (0)	0 (0)
**Females**		47 (51)	1471 (1500)	7 (14)	13 (15)
		3.1 (3.3)	95.6 (94.9)	0.5 (0.9)	0.8 (0.9)
	**Age (years)**				
	< 30	4 (4)	158 (162)	1 (3)	2 (2)
	30–40	4 (4)	91 (93)	0 (0)	2 (2)
	41–50	9 (10)	134 (138)	1 (3)	1 (3)
	51–60	13 (15)	164 (175)	3 (6)	3 (3)
	61–70	5 (5)	226 (232)	0 (0)	2 (2)
	71–80	5 (6)	320 (321)	1 (1)	2 (2)
	81–90	5 (5)	307 (308)	1 (1)	1 (1)
	91–100	2 (2)	68 (68)	0 (0)	0 (0)
	>100	0 (0)	3 (3)	0 (0)	0 (0)
**Test period**					
1: 01.03.20–01.06.20		8 (8)	807 (820)	3 (3)	3 (3)
		6.3 (5.4)	25.6 (25.6)	18.6 (13.0)	12.5 (11.1)
2: 02.06.20–01.09.20		11 (11)	966 (974)	0 (0)	1 (1)
		8.7 (7.4)	30.7 (30.4)	0 (0)	4.2 (3.7)
3: 02.09.20–01.12.20		43 (43)	1008 (1025)	0 (0)	5 (5)
		33.9 (28.9)	32.0 (32.0)	0 (0)	20.8 (18.5)
4: 02.12.20–01.04.21		65 (87)	367 (386)	13 (20)	15 (18)
		51.2 (58.4)	11.7 (12.0)	81.3 (87.0)	62.5 (66.7)

**Table 2:** Comparison of positive and negative test results of the pharyngeal swab and the tracheal secretion.

*n*: Number of Patients (Number of Tests)					
Type of Test		Answer	Pharyngeal Swab		Total
			Positive	Negative	
**Tracheal secretion**		Positive	127 (149)	24 (27)	151 (176)
		Negative	16 (23)	3148 (3205)	3164 (3228)
Total			143 (172)	3172 (3232)	3315 (3404)

**Table 3:** Overview of the tentative diagnoses at admission and code of the tentative diagnoses of patients who were registered with discordant test results. Only registered tentative diagnoses associated to discordant test results are included in the table.

Discordance COVID-19 Tests vs Tentative Diagnoses	Number of Patients Registered with (% of Registered Specific Tentative Diagnoses)	
Tentative Diagnose at Admission	The Diagnosis Code in Total	Discordant Test Results
DB972A: COVID-19—severe acute respiratory syndrome	38	12 (31.6)
DB342A: COVID-19 without location definition	137	15 (10.9)
DZ038PA1: COVID-19 suspicion	258	1 (0.4)
DB948A: Post-COVID-19 condition	4	1 (25.0)
DJ189: Pneumonia	331	1 (0.3)
DR509: Fever	249	2 (0.8)
DI460: Cardiac arrest with successful resuscitation	33	1 (3.0)
DA449: Bacterial infection	44	1 (2.2)
DA419: Sepsis	28	1 (3.6)
DT659: Intoxication	5	1 (20.0)
DR060: Dyspnea	565	1 (0.2)
DI330: Infectious endocarditis	6	1 (16.7)
DZ039: Suspicion of disease	6	1 (16.7)
DR992: Cardiac death	5	1 (20.0)
**Total**	1378	40

## Data Availability

Original data can be provided from corresponding author upon request.
